# Mechanisms of chromatin remodeling by the human Snf2-type ATPase SNF2H

**DOI:** 10.1038/s41422-025-01103-w

**Published:** 2025-04-03

**Authors:** Deepshikha Malik, Ashish Deshmukh, Silvija Bilokapic, Mario Halic

**Affiliations:** Department of Structural Biology, St. Jude Children’s Research Hospital, Memphis, TN USA

**Keywords:** Cryoelectron microscopy, Chromatin remodelling, Nucleosomes

Dear Editor,

Chromatin remodelers play a crucial role in the organization of chromatin. All known remodeler enzymes use an Snf2-type ATPase motor to slide nucleosomal DNA around the histone octamer,^1,2^ but the precise underlying mechanism is unclear. Here, we use cryo-electron microscopy (cryo-EM) to visualize the continuous motion of nucleosomal DNA induced by human chromatin remodeler SNF2H, an ISWI family member.^1,3,4^

Interactions between chromatin remodelers and nucleosomes have been extensively studied by cryo-EM on cross-linked complexes,^5–9^ which showed remodelers in different nucleotide-bound states.^7–11^ In particular, Snf2 and ISWI were captured bound to non-hydrolyzable ADP-BeFx, to ADP, or in their nucleotide-free forms. The ADP-BeFx-bound structures showed the nucleosome in a near-canonical conformation, representing a state prior to ATP hydrolysis and DNA translocation.^7,8,11^ By contrast, the ADP-bound structures showed a 1-bp bulge in the DNA at superhelix 2 (SHL2), suggesting a post-hydrolysis state^7,8,12^; moreover, the DNA tracking strand showed a distortion from the entry site to SHL2,^7,8^ suggesting that the two DNA strands are translocated asynchronously, but no intermediate states or guide strand movement have been captured so far.^3,4^

To address these gaps, we present a set of cryo-EM structures of human SNF2H actively remodeling nucleosomes. We purified human SNF2H and reconstituted a nucleosome with an 80 bp of linker DNA positioned on one side (Supplementary information, Fig. S1a). The purified SNF2H bound to the nucleosome and remodeled it upon activation by ATP (Supplementary information, Fig. S1b, c). We collected cryo-EM images of the ATP-activated complex frozen at 5 s, 2 min or 10 min after the addition of ATP and at two MgCl_2_ concentrations without cross-linking (Supplementary information, Fig. S1d, e). We merged the datasets from all conditions and performed extensive data analyses to determine 13 unique structures of the active SNF2H bound to the nucleosome (Supplementary information, Fig. S2 and Table S1). The individual datasets, collected at different time-points and MgCl_2_ concentrations, showed the same conformations of the SNF2H–nucleosome complex, with small variations in the fraction of particles in each conformation (Supplementary information, Fig. S3a), likely because the complex undergoes multiple cycles of DNA translocation that are not synchronized.

In agreement with previous structures,^7,11,13^ SNF2H binds to the nucleosome at SHL2 (Supplementary information, Fig. S2d). Different DNA sequences are likely located at SHL2 at any given time point, indicating that the mechanisms of DNA translocation are not sequence-specific. In each structure, we observed resolved density for amino acid side chains and DNA bases in the nucleosome, and for SNF2H ATPase domain side chains and nucleotide density in the active site in most conformations (Supplementary information, Fig. S3b, c).

Based on the conformations of DNA at SHL2 and of SNF2H, we clustered our 13 structures into five groups (A to E) (Fig. 1a; Supplementary information, Fig. S4a). Groups A to D showed all components at high resolution; group E showed a flexible SNF2H at low resolution. Since DNA translocation is a directional movement, we were able to establish the relative order of the different structures (Supplementary information, Fig. S4a, b).

**Fig. 1:**
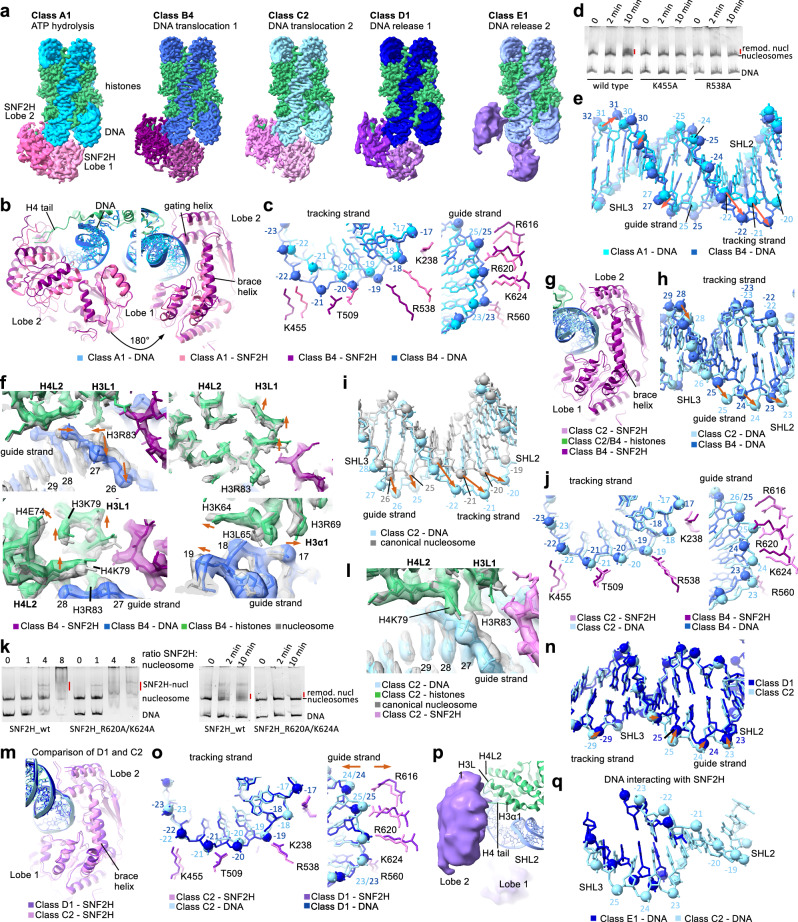
Cryo-EM structures of active human SNF2H bound to the nucleosome. **a** Nucleosome–SNF2H complexes during ATP hydrolysis (class A1), tracking strand movement (class B4), guide strand movement (class C2), DNA release state 1 (class D1), and DNA release state 2 (class E1). **b**, **c** Overlay of class B4 and A1 structures, showing changes in SNF2H (**b**) and in SNF2H–DNA interactions (**c**). Nucleotide numbering is relative to the dyad; negative numbers mark the tracking strand, and positive numbers mark the guide strand. **d** Native gel stained for DNA showing nucleosome remodeling by wild-type and mutant SNF2H. **e** Overlay of the nucleosomal DNA at SHL2 and SHL3 in class B4 and A1 structures. Orange arrows indicate DNA phosphate group movements. **f** Overlay of class B4 and canonical nucleosome structures. Orange arrows indicate histone residue movements. **g**, **h** Overlay of class C2 and B4 structures, showing conformational changes in SNF2H (**g**) and DNA (**h**). **i** Overlay of the DNA at SHL2 and SHL3 in class C2 and the canonical nucleosome structures. **j** Overlay of class C2 and B4 structures, showing changes in SNF2H interaction with DNA tracking (left) and guide (right) strands. **k** Native gel stained for DNA showing nucleosome binding (left) and remodeling (right) by wild-type and R620A K624A mutant SNF2H. **l** Overlay of class C2 and canonical nucleosome structures showing changes in histones. **m** Overlay of class D1 and C2 structures showing conformational changes in SNF2H. **n** Overlay of the DNA at SHL2 and SHL3 in class D1 and C2 structures. **o** Overlay of class D1 and C2 structures showing SNF2H–DNA interactions. **p** Class E structure showing interactions of SNF2H with DNA and histones. **q** Overlay of the DNA at SHL2 and SHL3 in class E1 and C2 structures; nucleotides that interact with SNF2H in each class are shown.

In group A (ATP hydrolysis, 15% of A-D particles) (Supplementary information, Figs. S3a, S5), DNA and SNF2H adopt a conformation overall similar to that in the structures of nucleosome-remodeler complexes with ADP-BeFx.^11^ The DNA at SHL2 is pulled out by 1–2 Å relative to the canonical nucleosome structure (Supplementary information, Fig. S5c, d) and the density for nucleotide bound to SNF2H is in a position similar to that of ADP-BeFx^11^ (Supplementary information, Fig. S5e), which is consistent with either ATP or ADP-Pi occupancy. However, we observe a gradual rotation of SNF2H lobe 2 towards SHL3, relative to the ADP-BeFx-bound structure,^7^ starting in the A1 structure (Supplementary information, Fig. S5a, b) and becoming more pronounced in the A2 and A3 structures (Supplementary information, Fig. S5f, g). These features indicate that group A structures represent conformational states downstream of the ADP-BeFx-bound state.

In group B (tracking strand movement, 45% of A-D particles) (Supplementary information, Figs. S3a, S6), the DNA tracking strand is pulled out more than the guide strand, deforming DNA locally at SHL2 and SHL3. SNF2H adopts a canonical open conformation, identical to that of ADP-bound ISWI (Supplementary information, Fig. S6b) and with similar density for the bound nucleotide (Supplementary information, Fig. S6a),^7,8^ indicating that group B structures represent post-ATP hydrolysis states (Supplementary information, Fig. S6a). However, the position of SNF2H relative to the nucleosome is different from the ADP-bound structures (Supplementary information, Fig. S6a). Compared to A3, the SNF2H lobe 2 in the B1 structure continues to rotate toward SHL3, whereas the brace helix and lobe 1 remain stable (Supplementary information, Fig. S6c, d). The other group B structures show gradual conformational transitions in SNF2H: relative to the B1 structure, SNF2H moves further outward in the B2–B4 structures, with the brace helix moving away from the nucleosome (Supplementary information, Fig. S6e, f).

By overlaying A1 and B4 structures, we can visualize the extent of lobe 2 rotation toward SHL3 and the outward rotation of the brace helix (Fig. 1b). Lobe 1 rotates in the same direction as lobe 2, but its rotation is more limited (Supplementary information, Fig. S6g), and the interactions between the guide strand and lobe 1 or the brace helix are not altered (Fig. 1c). By contrast, the movements of lobe 2 alter some of its interactions with the DNA tracking strand (Fig. 1c). For example, T509 interacts with the nucleotide at position –21 in group A and at position –22 in group B structures (nucleotide numbering is relative to the dyad; negative numbers mark the tracking strand). Likewise, R538 interacts with the nucleotide at –18 in group A and at –19 in group B structures. Several lobe 2 residues interact with the same nucleotide in group A and B structures, such as K455 with nucleotide at –22. We assessed the importance of the interactions between lobe 2 residues and tracking strand, by generating SNF2H R538A or K455A mutants. Those mutations severely reduced the chromatin remodeling activity of SNF2H, while showing a small impact on its ATPase or nucleosome binding activity (Fig. 1d; Supplementary information, Fig. S6h–j).

The lobe 2 movements pull the nucleosomal DNA away from the histone octamer surface at SHL2 and SHL3, with both guide and tracking DNA strands displaced outward but to different extents: the tracking strand is pulled out by ~6 Å in group B structures, while the guide DNA strand is pulled out by ~1–2 Å in B1 and ~3–4 Å in B4 structure (Fig. 1c, e; Supplementary information, Fig. S7a–d), concomitantly with the gradual outward movement of the brace helix. From the entry site up to SHL3, both DNA strands adopt the canonical conformation but they advance by 1 bp compared to their position in the canonical nucleosome (Supplementary information, Fig. S7e). At SHL2 and SHL3, only the tracking strand is pulled by one nucleotide (Fig. 1e), whereas sliding of the guide strand is blocked by the brace helix (Fig. 1b). The dissimilarity in the tracking and guide strand movements results in DNA backbone deformation and base tilting at SHL2 and SHL3, which is necessary to accommodate the 1-nt difference between guide and tracking strands (Fig. 1e; Supplementary information, Fig. S7c, d). The observed DNA distortion and formation of short A-DNA helix at SHL2 in group B are similar to features seen in the nucleosome bound to chromatin remodeler Chd1 in a nucleotide-free state.^5^

In the B4 structure, the DNA phosphate groups and bases are in an intermediate position, in between two canonical positions (Supplementary information, Fig. S7f). To accommodate this positioning and local distortion of the nucleosomal DNA, histones H3 and H4 undergo rearrangements (Fig. 1f; Supplementary information, Fig. S7g), with several alterations in residues in loops H3 L1 (E76–S86) and H4 L2 (A76–T80), which interact with DNA at SHL2.5. For example, the nucleotide 28 in B4 is in a position between those of nucleotides 27 and 28 in the canonical nucleosome. This DNA change induces an inward flipping of H3R83, which interacts with DNA phosphate at position 28 in B4 structure, instead of position 27 as in canonical nucleosome structures (Fig. 1f). The DNA phosphate at position 28 moves closer to histones and pushes H4K79 toward the histone core. Thus, accommodations to DNA changes lead to the movement of the entire H3 L1 and H4 L2 loops, also affecting residues that do not interact with DNA, such as H4E74 and H3K79. We also observe rearrangements of H3K64 and H3L65 side chains in the H3 α1 helix, which interacts with DNA at SHL1.5 (Supplementary information, Fig. S7g). Such histone rearrangements are not observed on the nucleosome side, where SNF2H is not bound (Supplementary information, Fig. S7h). To test the importance of histone adaptation for nucleosome remodeling, we non-specifically cross-linked histones with glutaraldehyde and found that SNF2H cannot mobilize nucleosomes containing cross-linked histones (Supplementary information, Fig. S7i–k). These findings reconcile conflicting observations regarding histone conformational changes during chromatin remodeling^14^ (Supplementary Note). The histone backbone changes we observe are relatively small (1–2 Å), but larger structural changes may occur more transiently.

In group C (guide strand movement, 30% of A-D particles) (Supplementary information, Figs. S3a, S8), the direction of SNF2H movement changes compared to group B structures: SNF2H rotates downward and outward, away from the histone octamer, while the brace helix continues its outward movement (Fig. 1g). SNF2H adopts the canonical open conformation observed in group B, and moves as a rigid body relative to the nucleosome (Supplementary information, Fig. S8a). These movements gradually pull the guide strand of DNA outward at SHL3 and correct the distortion at SHL2 and SHL3 seen in group B structures (Fig. 1h), while preserving the interactions between SNF2H residues and DNA (Fig. 1j; Supplementary information, Fig. S8c–j). Both DNA strands are displaced outward more than 6 Å, relative to the canonical nucleosome, taking a longer path that accommodates one additional base pair at SHL2 (Fig. 1i); thus, the nucleosomal DNA is effectively moved by 1 bp from the entry site to SHL2 (Supplementary information, Fig. S8b). Mutating brace helix residues markedly reduced the chromatin remodeling activity of SNF2H (Fig. 1k; Supplementary information, Fig. S8k, l), supporting their essential role in DNA translocation.

The DNA rearrangements in group C structures relieve most of the histone distortions seen in group B structures, though some distortions remain, since the DNA is still pulled away from the histone octamer surface compared to the canonical nucleosome (Fig. 1l; Supplementary information, Fig. S8m). For example, the nucleotide in position 28 completes its movement to position 27 in C2. Consequently, H3R83, which interacted with DNA at position 28 in group B structures, moves back to its canonical conformation in C2 and interacts with DNA at position 27 (Fig. 1l).

Our structures indicate that SNF2H releases DNA in two steps, by the gradual opening of the two lobes and brace helix movements. In group D (guide strand release, 10% of A-D particles) (Supplementary information, Figs. S3a; S9), the DNA starts moving back toward the histone octamer surface. Both SNF2H lobes continue their outward movement, away from SHL3 and toward SHL2, but lobe 2 also moves upward and away from lobe 1 (Supplementary information, Fig. S9a–c). Despite the opening of the lobes, density consistent with ADP can still be observed between them (Supplementary information, Fig. S3c). The brace helix continues its outward movement, away from the nucleosome, weakening its interaction with the guide strand and allowing the DNA to move 1–2 Å back toward the surface of the histone octamer (Fig. 1n, o; Supplementary information, Fig. S9d–g). Together, these observations indicate that the opening of two lobes and brace helix movement lead to DNA release from SNF2H.

In the two group E structures, the two lobes of SNF2H are fully separated, suggesting that the nucleotide has dissociated. Each lobe remains flexibly bound to the nucleosome (Fig. 1a, p; Supplementary information, Fig. S9g); lobe 2 continues its upward movement toward histones and releases the DNA at SHL2, which returns to the conformation in the canonical nucleosome (Fig. 1q). In the E1 structure, lobe 2 maintains its interactions with histones H3 and H4 (Fig. 1p) and with DNA near SHL3 (Fig. 1q; Supplementary information, Fig. S9h).

In summary, our 13 structures illustrate the continuous motions of DNA, histones, and SNF2H during chromatin remodeling. Conformational changes in SNF2H induced by ATP hydrolysis pull the nucleosomal DNA at SHL2 in two steps (from A to B and from B to C structures), resulting in the formation of a DNA bulge that accommodates one additional base pair at SHL2. Subsequently, the SNF2H lobes open and partially release the DNA at SHL2 (C to D structures), followed by full separation of the two lobes and complete DNA release (group E) (Supplementary information, Fig. S10a and Videos S1–S4). Notably, the SNF2H residues that contact DNA at SHL2 during these steps are conserved across chromatin remodeler families, suggesting that the mechanisms we describe for SNF2H likely apply to all ATP-dependent chromatin remodeling enzymes (Supplementary information, Fig. S10b).

The majority of our particles (75%) belong to groups B and C, indicating that the enzyme spends most of its time in those states. This is consistent with Förster resonance energy transfer experiments,^15^ which revealed a delay between DNA movement at the entry site (class B1) and translocation toward the exit site (after class D1).

While we interpret the group E structures as states during the DNA release step, they could correspond to the initial association of SNF2H with the nucleosome, its dissociation from the nucleosome, or a regulatory state that does not occur at every DNA translocation cycle. Notably, in the E1 structure, SNF2H interacts with histones and DNA near SHL3, and these contacts could provide directionality to the DNA movement, as they would prevent the propagation of the bulge toward the entry site when the SHL2 DNA is released — thus, the additional 1 bp accumulated at SHL2 can only propagate toward the dyad.

## Supplementary information

Supplementary Information

Video 1

Video 2

Video 3

Video 4

## Data Availability

EM maps and models were deposited in the EMDB and PDB with accession codes: nucleosome (EMD-47425, PDB-9E1Y), nucleosome–SNF2H class A1 (EMD-47412, PDB-9E1L), A2 (EMD-47413, PDB-9E1M), A3 (EMD-47414, PDB-9E1N), B1 (EMD-47415, PDB-9E1O), B2 (EMD-47416, PDB-9E1P), B3 (EMD-47417, PDB-9E1Q), B4 (EMD-47418, PDB-9E1R), C1 (EMD-47421, PDB-9E1U), C2 (EMD-47422, PDB-9E1V), C3 (EMD-47423, PDB-9E1W), D1 (EMD-47424, PDB-9E1X), E1 (EMD-49267) and E2 (EMD-49268).
