# Supplementary material for: Mechanisms of chromatin remodeling by the human Snf2-type ATPase SNF2H
Source: Cell Res. 2025 Apr 3;35(6):465–8. doi: 10.1038/s41422-025-01103-w (PMC12134373; doi:10.1038/s41422-025-01103-w)
Supplement: Supplementary file 1 — Supplementary Information [file 41422_2025_1103_MOESM1_ESM.pdf]

Supplementary Information

Mechanisms of chromatin remodeling by an Snf2-type ATPase SNF2H

Supplementary Note

Previous structures had captured Snf2 and ISWI chromatin remodelers bound to non-hydrolyzable ATP analog ADP-BeFx [7,8,11]. Our group A structures show overall similarity to the ADP-BeFx bound structures, but we observe gradual rotation of lobe 2, suggesting that our structures represent states that occur during ATP hydrolysis or before Pi release. In the group B structures SNF2H shows overall similarity to structures of Snf2 or ISWI bound to ADP [7,8], but it adopts a different position relative to the nucleosome. The ADP-bound structures of ISWI and Snf2 showed asynchronous translocation of the tracking strand by 1 nucleotide from the entry site to SHL2. In contrast, our group B structures show both DNA strands translocating together from the entry site until SHL3, with asynchronous translocation occurring only at SHL2 and SHL3, where movement of guide strand is blocked by the brace helix. These differences suggest that the ADP-bound state described previously [7,8] might be a short-lived intermediate that exists between our groups A and B structures that we did not capture with actively translocating SNF2H. DNA distortion at SHL2 and SHL3 and formation of short A-DNA helix at SHL2 were previously observed in the structure of a nucleosome with chromatin remodeler Chd1 in a nucleotide-free state [5]. In agreement, our structures show a short A-DNA helix at SHL2 in group B structures, as reported by the modeling algorithm 3DNA [16].

Our group B structures also show that histones adjust to the DNA movements induced by SNF2H activity, which reconciles conflicting observations in the literature. A previous report showed that cysteine substitutions and cross-linking of residues in H3 L1 (H3L82C) and H4 L2 (H4V81C) reduced SNF2H and ISW2 activity, indicating that structural alterations in histones are important for chromatin remodeling [14,17]. However, histone conformational changes had not been observed in previous structures of chromatin remodelers stabilized by different nucleotides [18,19]. The intrinsic plasticity of histones was previously described: we reported spontaneous histone deformation around SHL2 in nucleosomes without any remodelers [20,21]; dynamic behavior of histones has been observed in recent MD simulations [22]. Our cryo-EM structures of SNF2H actively remodeling the nucleosome show that residues in histones H3 and H4 undergo conformational changes (both at backbone and side chain level) in order to maintain their interactions with moving DNA phosphates. The histone backbone changes we observe are relatively small (1-2 Å) and although both residues H3L82 and H4V81 rearrange in B4 structure, the distance between them remains similar to the canonical nucleosome structure, suggesting that cross-linking those residues might underestimate functional importance of conformational changes in histones [18,19]. However, larger structural changes may occur more transiently, and it is possible that cross-linking those positions might disrupt larger motions of H3 L1 and H4 L2 that occur during DNA translocation.

Methods

Histone expression and purification

All the *Xenopus laevis* histones were separately over-expressed in BL21(DE3) pLysS bacterial strain and purified from inclusion bodies, as previously described [23].

The transformed cells were grown at 37 °C and induced with 1 mM IPTG at OD_{600} of 0.6. After 3h of induction, the cells were harvested, resuspended in lysis buffer (50 mM Tris-HCl (pH-7.5), 150 mM NaCl, 1 mM EDTA, 1 mM DTT and 0.1 mM PMSF), and frozen. The frozen cells were thawed and sonicated. The pellet containing inclusion bodies was recovered by centrifugation at 35,000 rcf for 20 min at 4 °C. The recovered pellet was washed two times with lysis buffer containing 1% Triton X-100 followed by two times wash with lysis buffer without Triton X-100. The pellet, containing histones as in-soluble inclusion bodies was retrieved by centrifugation after each washing step.

The inclusion body pellet for each histone protein was extracted in a buffer containing 50 mM Tris (pH 7.5), 2 M NaCl, 6 M guanidine hydrochloride and 1 mM DTT for overnight at room temperature. Any in-soluble components were removed by centrifugation. Histone pairs (H2A-H2B and H3-H4) were com-bined in equimolar ratios and dialyzed twice in 1L of refolding buffer (25 mM HEPES/NaOH (pH 7.5), 2 M NaCl and 1 mM DTT) at 4 °C. Precipitates were removed by centrifugation for 20 minutes at 13,000 rpm at 4 °C. The soluble histone pairs were purified by cation-exchange chromatography in batch (SP Sepharose Fast Flow resin). The samples were diluted fourfold with buffer without salt (25 mM HEPES/NaOH (pH 7.5) and 1 mM DTT) and bound to the resin for 30 min. The resin was extensively washed with 500 mM salt buffer in batch (25 mM HEPES/NaOH (pH 7.5), 500 mM NaCl and 1 mM DTT) and loaded onto a disposable column. On the column, the resin was washed, and pure proteins were eluted with 25 mM HEPES/NaOH (pH 7.5), 2 M NaCl and 1 mM DTT. Soluble histone pairs were concentrated and purified on a Superdex S200 size-exclusion column (GE) equilibrated in 25 mM HEPES/NaOH (pH 7.5), 2 M NaCl and 1 mM DTT. Clean protein fractions were pooled, concentrated and flash-frozen.

Histone octamer preparation

Histone octamer purification was done using the following protocol [23,24]. In short, a 2-fold molar excess of the H2A-H2B dimer was mixed with the H3-H4 tetramer in the presence of buffer IV containing 2 M NaCl, 25 mM HEPES (pH 7.5), and 1 mM DTT. After overnight incubation at 4 °C, octamer was sepa-rated from excess dimer using a Superdex S200 Increase 10/300 GL column on AKTA FPLC equili-brated with buffer IV. The fractions of interest were analyzed on SDS-PAGE, pooled, and concentrated for final nucleosome assembly.

Nucleosomal DNA preparation

Nuclesomal DNA was prepared by PCR amplification from a plasmid with 145 base pairs of 601 DNA sequeunce [25]. Primers were designed such that the final DNA product had a 80 base pair long overhang on one side of 601 DNA. The sequence of 601 with 80 bp of flanking DNA is as following:

GCACAGGATGTATATATCTGACACGTGCCTGGAGACTAGGGAGTAATCCCCTTGGCGGT-
TAAAACGCGGGGGACAGCGCGTACGTGCGTTTAAGCGGTGCTAGAGCTGTCTACGAC-
CAATTGAGCGGCCTCGGCACCGGGATTCTCCAGGGCGGCCGCGTATAGGGTCCATCA-
CATAAGGGATGAACTCGGTGTGAAGAATCATGCTTTCCTTGGTCATTAGGATCC

Nucleosome assembly

Nucleosome assembly was done by double bag dialysis as previously described [26]. Nucleosomal DNA was suspended in 25 mM HEPES/NaOH pH 7.5, 1 mM DTT, and 2 M NaCl. The histone octamer was mixed with DNA in equimolar ratios and placed into a dialysis button (membrane cutoff: 3.5 kDa MW). These buttons were then placed in a dialysis bag (6-8 kDa cut-off membrane) filled with 50 ml of buffer containing 25 mM HEPES (pH 7.5), 2 M NaCl and 1 mM DTT. The dialysis bag was immersed into 1 l of buffer containing 25 mM HEPES (pH 7.5), 1 M NaCl and 1 mM DTT and dialyzed overnight at 4 °C. After overnight dialysis, the 1L buffer was replaced with 1L of 25 mM HEPES (pH 7.5), 50 mM NaCl, and 1 mM DTT for a 6 hours dialysis. Finally, the last dialysis is done with 25 mM HEPES (pH 7.5) and 1mM DTT for an hour. The quality of nucleosome assemblies was checked on a 6% native PAGE which is run on 1x TBE, 200V at 4 °C. The gels were visualized on a Chemidoc.

Purification of SNF2H

SNF2H cloned in pET duet vector was ordered from GenScript. *E. coli* LOBSTR Rosetta bacterial culture, containing pET duet vector with SUMO tag on one site followed by SNF2H gene, was grown in TB medium containing the appropriate antibiotics at 37 °C until the optical density reached 0.6 at 600 nm. The culture was induced with 0.4 mM IPTG overnight at 16 °C. The cells were harvested and frozen at -20 °C. For purification, frozen pellets were thawed and resuspended in lysis buffer (20 mM Tris-HCl/ HEPES pH 8.0, 2 M NaCl, 1 mM PMSF, 10 mM Imidazole, 1 mM DTT) and lysed. The cleared supernatant was incubated with pre-equilibrated Ni Sepharose 6 Fast-flow resins. On the column, the resin was washed with 5 bed volumes of washing buffer I (20 mM Tris-HCl/HEPES pH 8.0, 2 M NaCl, 10 mM Imidazole 1 mM DTT), II (20 mM Tris-HCl pH 8.0, 150 mM NaCl, 75/50 mM Imidazole, 1 mM DTT) and III (20 mM Tris-HCl/HEPES pH 8.0, 150 mM NaCl, 300/500 mM Imidazole, 1 mM DTT). The bound protein was eluted with 5-bed volumes of buffer III and 5 column volume of 1M imidazole. The Ni purified protein was then purified on SP Sepharose cation exchange resins using 20 mM Tris-HCl/HEPES pH 8.0, 1 M NaCl and 1 mM DTT as the elution buffer. The protein was further treated with PreScission Protease, followed by overnight dialysis with buffer containing 20 mM HEPES

pH 7.5, 300mM NaCl, and 1 mM DTT. The protein was further concentrated and applied on size exclusion Superdex 200 Increase 10/300 equilibrated in 20 mM HEPES/NaOH, pH 7.5, 150 mM NaCl, 1 mM DTT. The fractions with the protein were analyzed on SDS-PAGE and concentrated. The concentrated protein was flash-frozen and stored at -80 °C. SNF2H mutant variants were created using site directed mutagenesis and purified using the same protocol as described above for SNF2H(WT). Quantification of SNF2H variants was performed using SDS-PAGE for assays.

Nucleosome Cross-linking

Nucleosome cross-linking was done by incubating assembled nucleosomes with 0.1% glutaraldehyde for 6 minutes at room temperature [20,21] and quenching with 50 mM Tris/HCl pH 7. The cross-linked nucleosome was analyzed on SDS-PAGE gel.

Salt disassembly of the nucleosome

To test if DNA was cross-linked to histones, 7 µl of nucleosome sample (25 mM HEPES/NaOH pH 7.5, 1 mM DTT) was supplemented with the buffer containing NaCl to achieve different NaCl concentrations (0, 0.1, 0.2, 0.3, 0.5, 0.7, 1, and 1.5 M) [20,21]. These samples were then incubated at 25 °C for 30 minutes. 4% glycerol (final concentration) was added to the samples and analyzed using 5% native PAGE. The gel was stained with SYBR Gold.

SNF2H binding to nucleosome

The binding assay for SNF2H and nucleosome was performed at room temperature in 20 mM Tris (pH 7.5), 70 mM KCl, 1 mM AMPPNP, 5 mM $MgCl_2$, and 0.02% NP-40. Typically, 50 nM nucleosome (601+80) was incubated with different SNF2H at different concentrations (50 nM, 250 nM, 500 nM and 1 µM) as indicated and incubated for 40 minutes. The bound and unbound nucleosomes were separated on a 5% native polyacrylamide gel and stained with SYBr gold dye. The gels were imaged using Chemidoc.

To analyze the binding of SNF2H wild-type and mutants to the nucleosome, 4nM nucleosome and different concentrations of SNF2H (4 nM, 16 nM, and 32 nM) were used.

SNF2H-remodeling assay

Nucleosome remodeling was done with 30 nM nucleosomes and 60 nM (two-fold molar excess) of SNF2H. The assay was carried out at room temperature in 20 mM Tris (pH 7.5), 70 mM KCl, 1 mM ATP, 5 mM $MgCl_2$ and 0.02% NP-40. This assay was also carried out in presence of 0.25 mM or 0.5 mM $MgCl_2$ to check the remodeling activity under reduced Mg^{2+} concentrations. The reaction was stopped with an excess of ADP/AMP-PNP and 50 mM EDTA. The reaction products were loaded on 3-12% bis-tris acrylamide gels and the gels were visualized after SYBr gold staining on a Chemidoc.

This method of visualization was followed for all the remodeling assays unless stated otherwise.
To analyze the remodeling activity of SNF2H mutants, 4 nM of nucleosome and 4 nM of wt or mutant SNF2H were used. The assay was done as described above and the reaction products were analyzed on 5% native polyacrylamide gels. To check the remodeling of cross-linked nucleosomes, 4 nM of nucleosomes and 4 nM of SNF2H were used and reaction products were run on a 5% native polyacrylamide gel and visualized as described above.

ATPase assay

To measure the ATPase activity of SNF2H, ADP-glo™ kinase assay kit from Promega was used. This kit measures the amount of ADP released by an ATPase. 32 nM of SNF2h wild type or mutant was incubated with 4 nM of nucleosome at room temperature for 20 minutes, with the same buffer used in remodeling assays. Two-fold serial dilutions of the complex were incubated with 1 mM ATP. The signal was measured at 590 nm using POLstar omega from BMG Labtech.

Assembly of SNF2H-nucleosome complex for cryo-EM analysis and grid preparation

Nucleosomes, with the final concentration not exceeding 0.3 mM, were assembled by ‘double bag’ dialysis using the same steps as described in the "Nucleosome assembly" section. 2.5 μM SNF2H and 0.625 μM nucleosomes were mixed in such a way that the final salt concentration did not exceed 50 mM NaCl. The binding was done for 20 minutes at room temperature and monitored as described in the binding assay section except for Mg^{2+} concentrations. 3 μl of SNF2h-nucleosome complex was applied to freshly glow-discharged Quantifoil R1.2/1.3 holey carbon grid. For freezing, FEI Vitrobot Mark IV chamber was maintained at 95% humidity and temperature of +12 °C. After 3 s blotting time, grids were plunge-frozen in liquid ethane using the FEI Vitrobot automatic plunge freezer. SNF2H was frozen at 5’’, 2’ and 10’ after addition of ATP at two different Mg^{2+} concentrations, 0.25 mM and 0.5 mM. For the 5'' time point, 1 mM ATP was added to the sample that was already on the grid and the sample was immediately frozen. For 2’ and 10’ timepoints, 1 mM ATP was added prior applying to the grid.

Cryo-EM data collection, image processing and model building

Electron micrographs were recorded on FEI Titan Krios at 300 kV with a Gatan Summit K3 electron detector using EPU at the cryo-EM facility at St. Jude Children’s Research Hospital. The image pixel size was 0.6485 Å per pixel on the object scale. Data were collected in a defocus range of 14,000 – 30,000 Å with a total exposure of 60 e^{-} $Å^{-2}$. We collected ~20 000 images of the sample frozen 5 seconds after activation (0.25 mM Mg^{2+}), ~23 000 and 48 000 of the sample frozen 2’ after activation (0.25 mM and 0.5 mM Mg^{2+} respectively) and ~20 000 frames 10’ after activation. 5mM $MgCl_2$ condition resulted in high aggregation of nucleosomes on cryo-EM grid and was not used for data collec-

tion. Micrographs were aligned with the MotionCorr2 software using a dose filter [27,28]. The contrast transfer function parameters were determined using CTFFIND4 [29].

Particles were picked using TOPAZ in RELION software package [30,31]. Particles were binned and 2D class averages were generated in RELION. Inconsistent class averages were removed from further data analysis. The initial reference was filtered to 40 Å in RELION. C1 symmetry was applied during refinements for all classes. Particles were split into many datasets and refined independently, and the resolution was determined using the 0.143 cut-off (RELION auto-refine option). All maps were filtered to resolution using RELION with a B-factor determined by RELION. Particles containing SNF2H (class 2) were classified many times leading to 11 different structures that have overlapping particles due to continuous motion. Final structures are combined from 5 different classifications, which were done with different masks and using different parameters, which resulted in distinct grouping of particles that are in continuous motion. Unique classes from each classification were selected. All classes were refined only locally using individual mask for each class. Final maps were processed using EMReady [32]. Initial molecular models were built using Modelangelo and Coot [33,34]. The model of the nucleosome (Protein Data Bank (PDB): 6WZ5) was refined into the cryo-EM map in PHENIX [35,36]. The model of SNF2H bound to the nucleosome (PDB:6NE3) was either rigid body fitted using PHENIX or built into cryo-EM maps using Modelangelo, manually adjusted and rebuilt in Coot and refined in Phenix [13]. Visualization of all cryo-EM maps was done in Chimera [37].

References

1. Narlikar, G. J., Sundaramoorthy, R. & Owen-Hughes, T. Mechanisms and functions of ATP-dependent chromatin-remodeling enzymes. *Cell* **154**, 490–503 (2013).
2. Clapier, C. R., Iwasa, J., Cairns, B. R. & Peterson, C. L. Mechanisms of action and regulation of ATP-dependent chromatin-remodelling complexes. *Nat Rev Mol Cell Biol* **18**, 407–422 (2017).
3. Eustermann, S., Patel, A. B., Hopfner, K.-P., He, Y. & Korber, P. Energy-driven genome regulation by ATP-dependent chromatin remodellers. *Nat Rev Mol Cell Biol* **25**, 309–332 (2024).
4. Nodelman, I. M. & Bowman, G. D. Biophysics of Chromatin Remodeling. *Annu Rev Biophys* **50**, 73–93 (2021).
5. Nodelman, I. M. *et al.* Nucleosome recognition and DNA distortion by the Chd1 remodeler in a nucleotide-free state. *Nat Struct Mol Biol* **29**, 121–129 (2022).
6. Yuan, J., Chen, K., Zhang, W. & Chen, Z. Structure of human chromatin-remodelling PBAF complex bound to a nucleosome. *Nature* **605**, 166–171 (2022).
7. Yan, L., Wu, H., Li, X., Gao, N. & Chen, Z. Structures of the ISWI-nucleosome complex reveal a conserved mechanism of chromatin remodeling. *Nat Struct Mol Biol* **26**, 258–266 (2019).
8. Li, M. *et al.* Mechanism of DNA translocation underlying chromatin remodelling by Snf2. *Nature* **567**, 409–413 (2019).
9. Liu, X., Li, M., Xia, X., Li, X. & Chen, Z. Mechanism of chromatin remodelling revealed by the Snf2-nucleosome structure. *Nature* **544**, 440–445 (2017).
10. Chittori, S., Hong, J., Bai, Y. & Subramaniam, S. Structure of the primed state of the ATPase domain of chromatin remodeling factor ISWI bound to the nucleosome. *Nucleic Acids Res* **47**, 9400–9409 (2019).
11. Chio, U. S. *et al.* Functionalized graphene-oxide grids enable high-resolution cryo-EM structures of the SNF2h-nucleosome complex without crosslinking. *Nat Commun* **15**, 2225 (2024).
12. Winger, J., Nodelman, I. M., Levendosky, R. F. & Bowman, G. D. A twist defect mechanism for ATP-dependent translocation of nucleosomal DNA. *Elife* **7**, e34100 (2018).
13. Armache, J. P. *et al.* Cryo-EM structures of remodeler-nucleosome intermediates suggest allosteric control through the nucleosome. *Elife* **8**, e46057 (2019).

14. Sinha, K. K., Gross, J. D. & Narlikar, G. J. Distortion of histone octamer core promotes nucleosome mobilization by a chromatin remodeler. *Science* **355**, (2017).
15. Sabantsev, A., Levendosky, R. F., Zhuang, X., Bowman, G. D. & Deindl, S. Direct observation of coordinated DNA movements on the nucleosome during chromatin remodelling. *Nat Commun* **10**, 1720 (2019).
16. Li, S., Olson, W. K. & Lu, X.-J. Web 3DNA 2.0 for the analysis, visualization, and modeling of 3D nucleic acid structures. *Nucleic Acids Res* **47**, W26–W34 (2019).
17. Hada, A. *et al.* Histone Octamer Structure Is Altered Early in ISW2 ATP-Dependent Nucleosome Remodeling. *Cell Rep* **28**, 282-294.e6 (2019).
18. Gamarra, N. & Narlikar, G. J. Histone dynamics play a critical role in SNF2h-mediated nucleosome sliding. *Nat Struct Mol Biol* **28**, 548–551 (2021).
19. Li, L., Yan, L. & Chen, Z. Reply to: Histone dynamics play a critical role in SNF2h-mediated nucleosome sliding. *Nat Struct Mol Biol* **28**, 552–553 (2021).
20. Bilokapic, S., Strauss, M. & Halic, M. Structural rearrangements of the histone octamer translocate DNA. *Nat Commun* **9**, 1330 (2018).
21. Bilokapic, S., Strauss, M. & Halic, M. Histone octamer rearranges to adapt to DNA unwrapping. *Nat. Struct. Mol. Biol.* **25**, 101–108 (2018).
22. Armeev, G. A., Kniazeva, A. S., Komarova, G. A., Kirpichnikov, M. P. & Shaytan, A. K. Histone dynamics mediate DNA unwrapping and sliding in nucleosomes. *Nat Commun* **12**, 2387 (2021).
23. Luger, K., Rechsteiner, T. J. & Richmond, T. J. Preparation of nucleosome core particle from recombinant histones. *Meth. Enzymol.* **304**, 3–19 (1999).
24. Ivic N, Groschup B, Bilokapic S, & Halic M. Simplified Method for Rapid Purification of Soluble Histones. *Croatica chemica acta* **89**, 153–162 (2016).
25. Lowary, P. T. & Widom, J. New DNA sequence rules for high affinity binding to histone octamer and sequence-directed nucleosome positioning. *J. Mol. Biol.* **276**, 19–42 (1998).
26. Bilokapic, S. & Halic, M. Nucleosome and ubiquitin position Set2 to methylate H3K36. *Nat Commun* **10**, 3795 (2019).

27. Grant, T. & Grigorieff, N. Measuring the optimal exposure for single particle cryo-EM using a 2.6 Å reconstruction of rotavirus VP6. *Elife* **4**, e06980 (2015).
28. Zheng, S. Q. *et al.* MotionCor2: anisotropic correction of beam-induced motion for improved cryo-electron microscopy. *Nat. Methods* **14**, 331–332 (2017).
29. Rohou, A. & Grigorieff, N. CTFFIND4: Fast and accurate defocus estimation from electron micrographs. *J. Struct. Biol.* **192**, 216–221 (2015).
30. Zivanov, J. *et al.* New tools for automated high-resolution cryo-EM structure determination in RELION-3. *Elife* **7**, (2018).
31. Bepler, T. *et al.* Positive-unlabeled convolutional neural networks for particle picking in cryo-electron micrographs. *Nat Methods* **16**, 1153–1160 (2019).
32. He, J., Li, T. & Huang, S.-Y. Improvement of cryo-EM maps by simultaneous local and non-local deep learning. *Nat Commun* **14**, 3217 (2023).
33. Jamali, K. *et al.* Automated model building and protein identification in cryo-EM maps. *Nature* **628**, 450–457 (2024).
34. Emsley, P., Lohkamp, B., Scott, W. G. & Cowtan, K. Features and development of Coot. *Acta Crystallogr. D Biol. Crystallogr.* **66**, 486–501 (2010).
35. Bilokapic, S., Suskiewicz, M. J., Ahel, I. & Halic, M. Bridging of DNA breaks activates PARP2-HPF1 to modify chromatin. *Nature* **585**, 609–613 (2020).
36. Adams, P. D. *et al.* PHENIX: a comprehensive Python-based system for macromolecular structure solution. *Acta Crystallogr. D Biol. Crystallogr.* **66**, 213–221 (2010).
37. Meng, E. C. *et al.* UCSF ChimeraX: Tools for structure building and analysis. *Protein Sci* **32**, e4792 (2023).

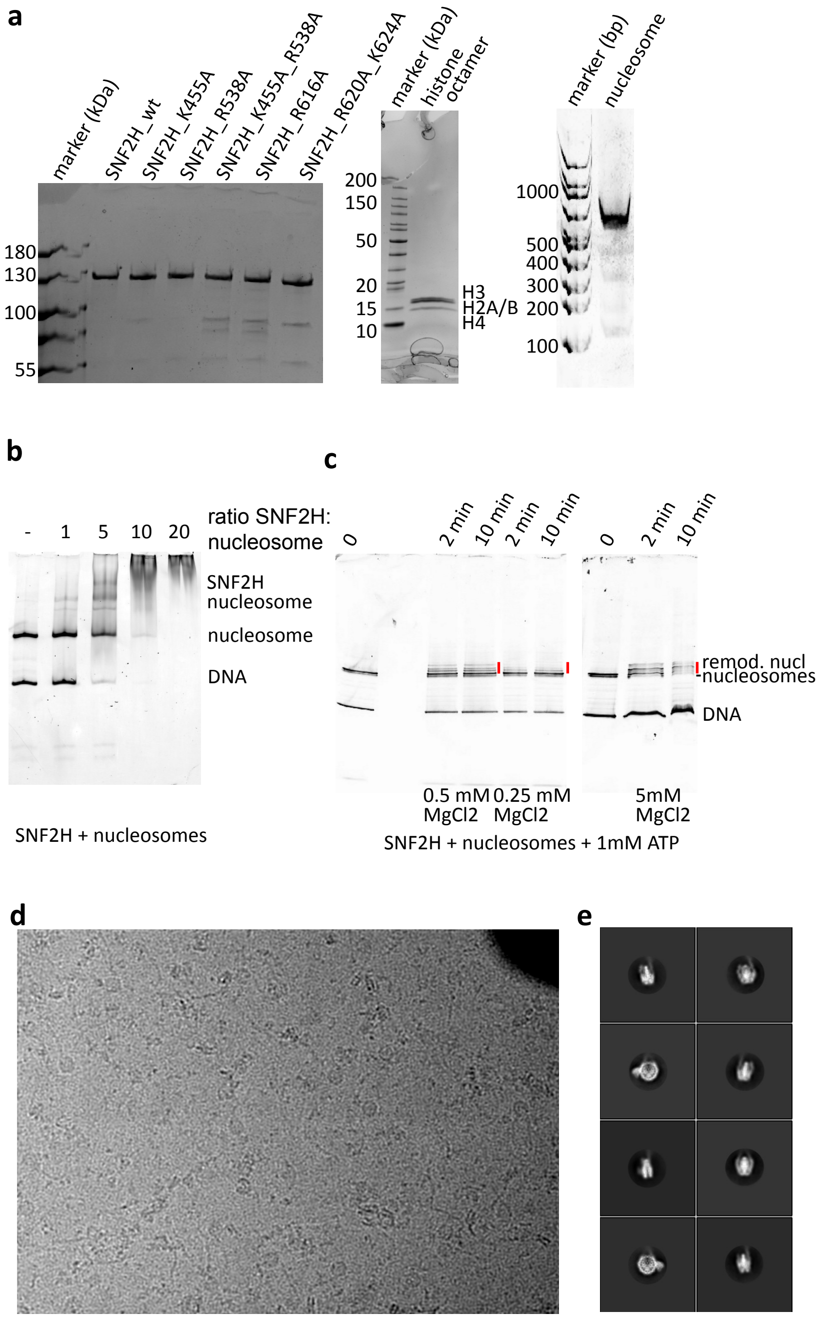

Figure S1. Assembly and EM analyses of SNF2H bound to nucleosome.

a) SDS-PAGE showing purified human SNF2H (left, wild type and mutants) and *Xenopus laevis* histone octamer assembly (middle). Right, native polyacrylamide gel stained for DNA shows the assembled nucleosome with 227 bp long DNA.

b) Native polyacrylamide gel stained for DNA showing binding of SNF2H to the nucleosome.

c) Native gel stained for DNA showing nucleosome remodeling by SNF2H upon addition of ATP.

d) Representative cryo-EM micrograph from a set of ~100,000 micrographs collected with Titan Krios electron microscope at 300 keV. Nucleosome particles in multiple orientations are visible.

e) Representative 2D class averages showing nucleosomes with SNF2H bound in different orientations.

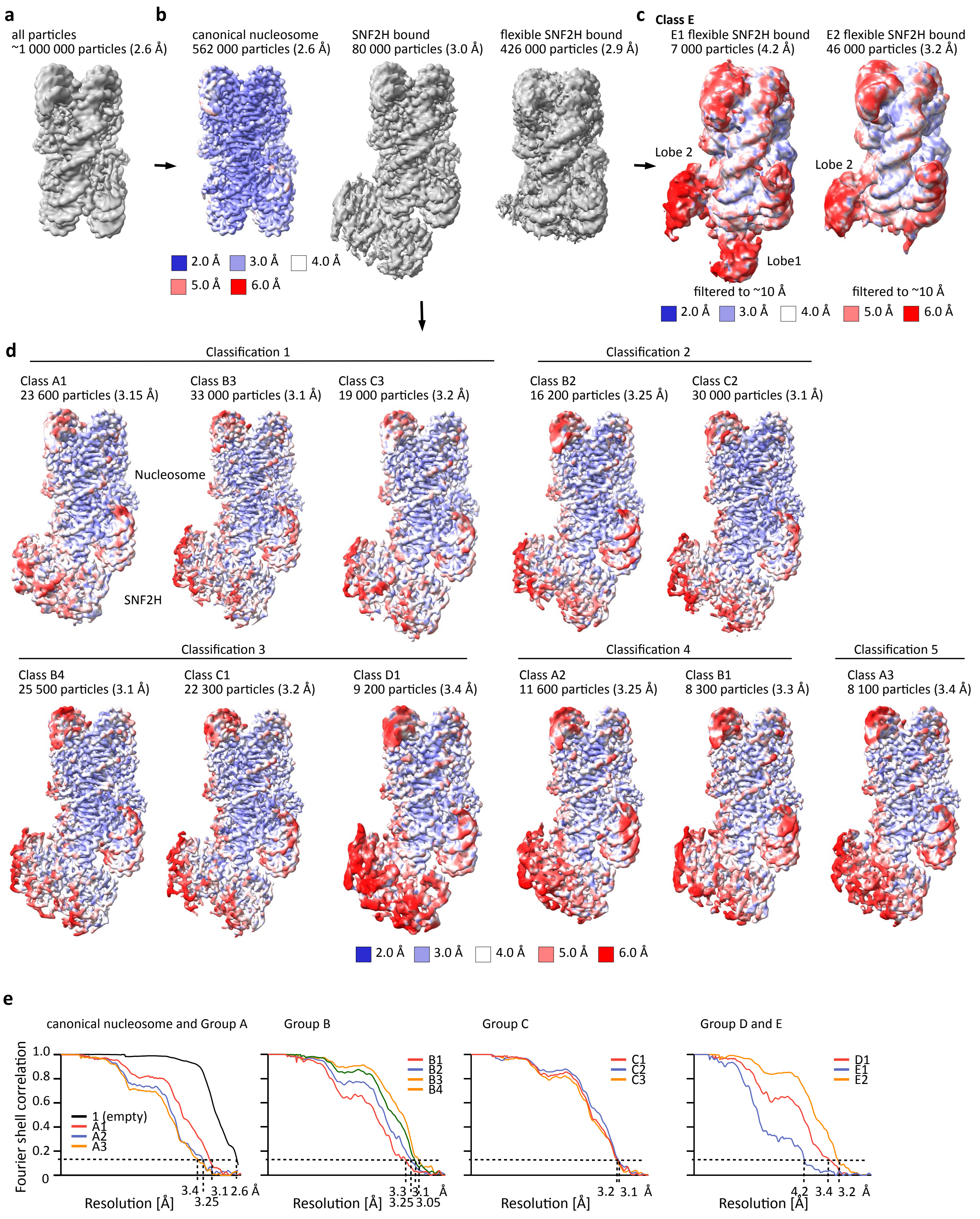
a
all particles
~1 000 000 particles (2.6 Å)
b
canonical nucleosome
562 000 particles (2.6 Å)
SNF2H bound
80 000 particles (3.0 Å)
flexible SNF2H bound
426 000 particles (2.9 Å)
2.0 Å 3.0 Å 4.0 Å 5.0 Å 6.0 Å
c
Class E
E1 flexible SNF2H bound
7 000 particles (4.2 Å)
E2 flexible SNF2H bound
46 000 particles (3.2 Å)
Lobe 2
Lobe1
Lobe 2
filtered to ~10 Å
filtered to ~10 Å
2.0 Å 3.0 Å 4.0 Å 5.0 Å 6.0 Å
d
Classification 1
Class A1
23 600 particles (3.15 Å)
Class B3
33 000 particles (3.1 Å)
Class C3
19 000 particles (3.2 Å)
Nucleosome
SNF2H
Classification 2
Class B2
16 200 particles (3.25 Å)
Class C2
30 000 particles (3.1 Å)
Classification 3
Class B4
25 500 particles (3.1 Å)
Class C1
22 300 particles (3.2 Å)
Class D1
9 200 particles (3.4 Å)
Classification 4
Class A2
11 600 particles (3.25 Å)
Class B1
8 300 particles (3.3 Å)
Classification 5
Class A3
8 100 particles (3.4 Å)
2.0 Å 3.0 Å 4.0 Å 5.0 Å 6.0 Å
e
canonical nucleosome and Group A
Group B
Group C
Group D and E
Fourier shell correlation
1.0 0.8 0.6 0.4 0.2 0
1 (empty)
A1
A2
A3
3.4 3.25 3.1 2.6 Å
Resolution [Å]
B1 B2 B3 B4
3.3 3.25 3.1 3.05 Å
Resolution [Å]
C1 C2 C3
3.2 3.1 Å
Resolution [Å]
D1 E1 E2
4.2 3.4 3.2 Å
Resolution [Å]

Figure S2. Classification of the SNF2H-nucleosome complex.

a) Cryo-EM map of nucleosome from the entire dataset, refined to 2.6 Å.

b) Classification of the data in panel a resulted in three major classes of nucleosomes: nucleosome alone (canonical), SNF2H-bound nucleosome and flexible SNF2H-bound nucleosome. The number of particles and overall resolution of each class are shown. The map of the canonical nucleosome is colored by local resolution.

c) Classification of flexible SNF2H-bound nucleosome data from panel b resulted in two classes, E1 and E2. The number of particles corresponding to each class and overall resolution are shown. Maps are colored by local resolution and filtered to ~10 Å using Gaussian filter in Chimera to visualize flexible SNF2H.

d) Classification of SNF2H-bound nucleosome from panel b. Because of the continuous motion of SNF2H, five different classifications were performed, resulting in 11 unique maps with overlapping particles. Unique maps from each classification are shown. Maps are colored by local resolution. The number of particles and resolution in each class are shown.

e) Fourier shell correlation (FSC) curves showing the resolution of the maps shown in panels a-d.

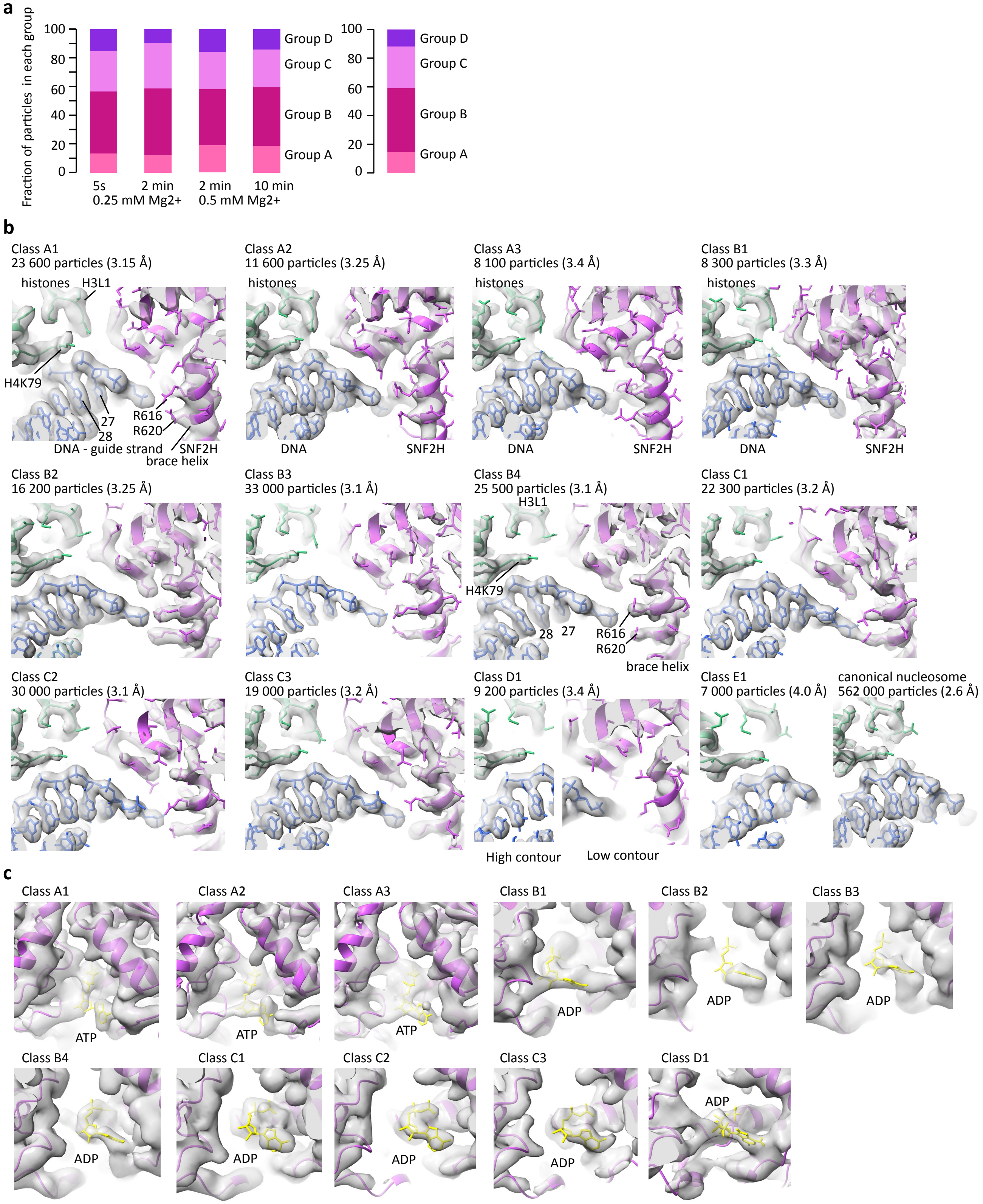
a
Fraction of particles in each group
100
80
60
40
20
0
5s
2 min
0.25 mM Mg2+
2 min
10 min
0.5 mM Mg2+
Group D
Group C
Group B
Group A
b
Class A1
23 600 particles (3.15 Å)
histones
H3L1
H4K79
27
28
R616
R620
DNA - guide strand
SNF2H
brace helix
Class A2
11 600 particles (3.25 Å)
DNA
Class A3
8 100 particles (3.4 Å)
Class B1
8 300 particles (3.3 Å)
Class B2
16 200 particles (3.25 Å)
Class B3
33 000 particles (3.1 Å)
Class B4
25 500 particles (3.1 Å)
Class C1
22 300 particles (3.2 Å)
Class C2
30 000 particles (3.1 Å)
Class C3
19 000 particles (3.2 Å)
Class D1
9 200 particles (3.4 Å)
High contour
Low contour
Class E1
7 000 particles (4.0 Å)
canonical nucleosome
562 000 particles (2.6 Å)
c
ATP
ADP

Figure S3. Distribution of particles and map quality of the SNF2H-nucleosome complex.

a) Left, bar charts showing fraction of particles in each group (A to D) in the datasets collected at different time points and $MgCl_2$ concentrations. Right, bar chart showing fraction of particles in each group in combined datasets (A-D).

b) A representative region showing map quality and fit of the model is shown for the nucleosome and SNF2H from each map. DNA bases (blue) and histone side chains (green) are well resolved in all maps. Side chains in SNF2H (magenta) are resolved in most maps.

c) The nucleotide-binding site of SNF2H in all classes from groups A to D, showing map quality and fit of the model. The density for bound nucleotide is shown in yellow.

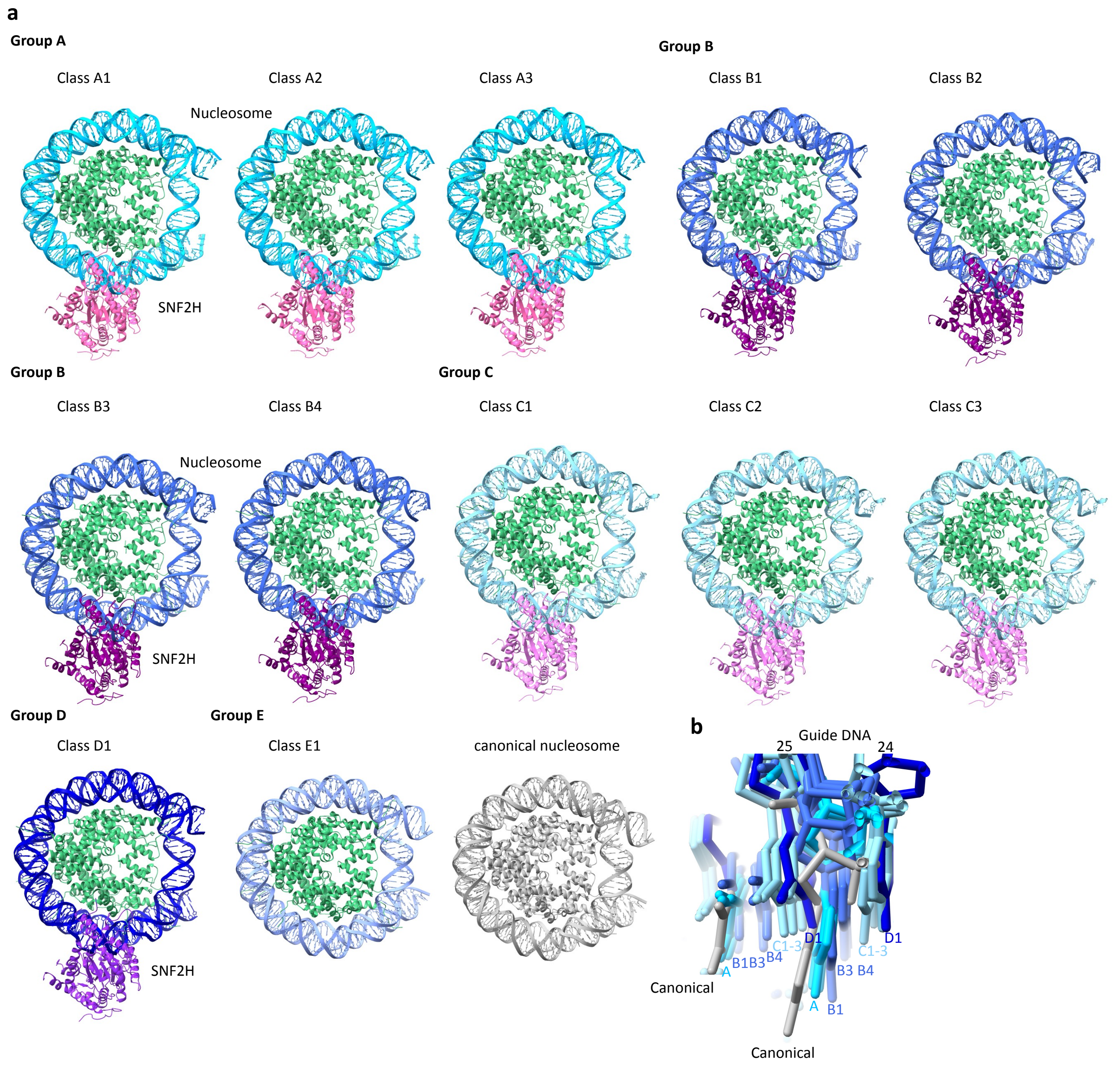

Figure S4. Models of SNF2H bound to nucleosome.

a) Models of SNF2H bound to nucleosome are shown for all structures. Based on DNA conformation and SNF2H conformation, the 13 structures were grouped into 5 groups.

b) Overlay of all models from groups A-D, showing the extent of the movement of nucleotides at positions 24 and 25 of the DNA guide strand.

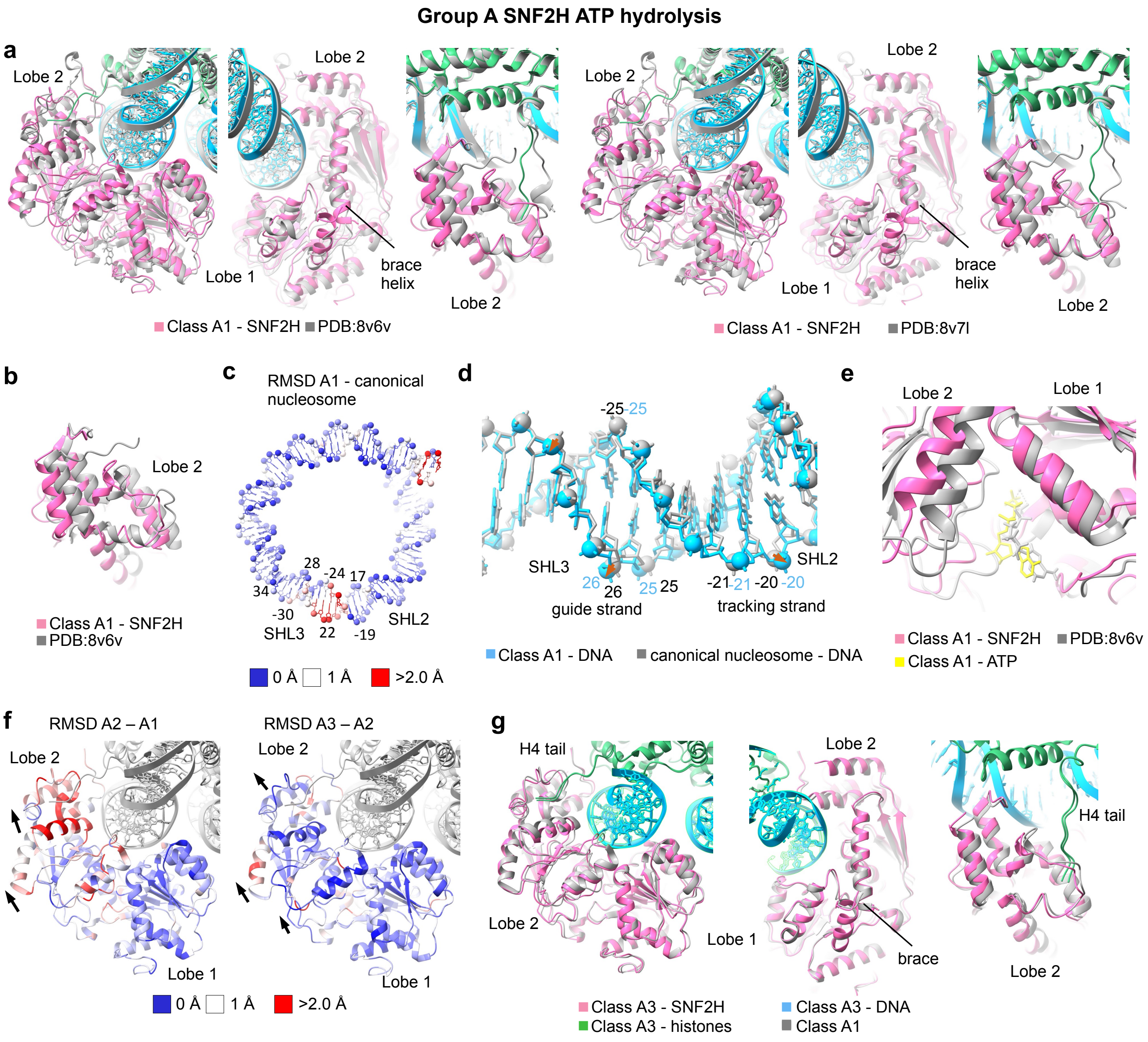

Figure S5. Conformational changes in SNF2H and nucleosome in group A

a) Close-up views of the overlay of class A1 (color) and two conformations of SNF2H–nucleosome complex with ADP-BeFx (grey; left, PDB 8v6v; right, PDB 8v7l). The structures were aligned on the nucleosome.

b) Close-up views of the overlay of SNF2H lobe 2 from class A1 (color) and nucleosome-SNF2H with ADP-BeFx (grey, PDB 8v6v) structures. The structures were aligned on SNF2H lobe 2.

c) RMSD of DNA between class A1 and canonical nucleosome structure, showing DNA changes upon SNF2H binding. Canonical nucleosomal DNA is shown.

d) Overlay of the DNA at SHL2 and SHL3 in class A1 (bright blue) and canonical nucleosome (grey) structures.

e) Close-up view of the overlay of SNF2H in class A1 (color) and ADP-BeFx-bound (grey, PDB:8v6v) structures, showing nucleotide-binding site.

f) RMSD of SNF2H between class A2 and A1 (left, A1 shown) and A3 and A2 (right, A2 shown) structures. Black arrows show direction of the movement.

g) Close-up views of the overlay of class A3 (color) and A1 (grey) structures, showing conformational changes in SNF2H.

Group B SNF2H DNA translocation 1

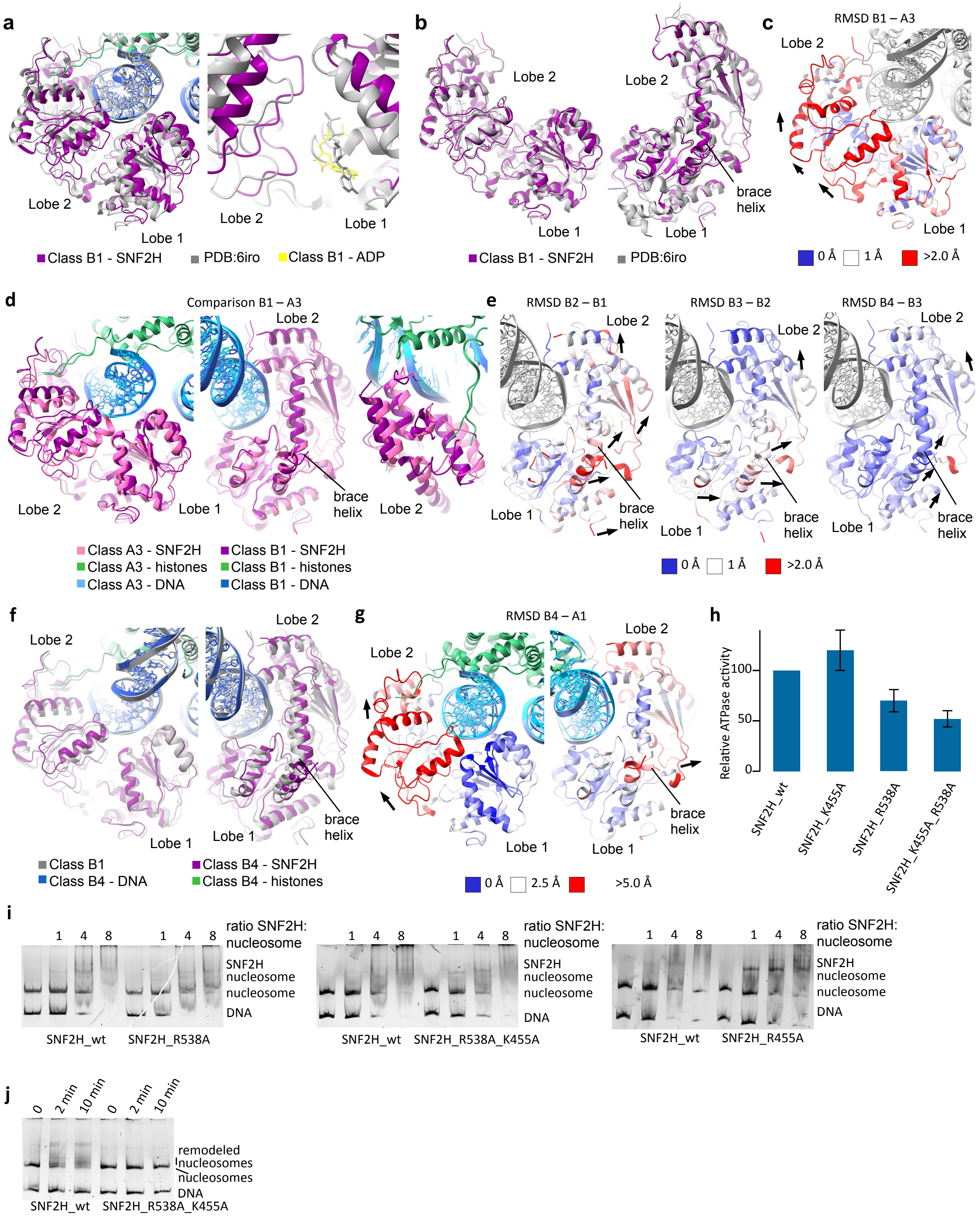

Figure S6. Conformational changes in SNF2H in group B

a) Close-up views of the overlay of class B1 (color) and nucleosome-SNF2H in ADP-bound state

(grey, PDB:6iro) structures, showing conformational changes in SNF2H (left) and nucleotide binding site (right). Nucleosome was used for alignment.

b) Close-up view of the overlay of SNF2H of class B1 (color) and SNF2H in ADP-bound state (grey, PDB:6iro) structures. SNF2H was used for alignment.

c) RMSD of SNF2H between class B1 and A3 structures. A3 structure is shown. Black arrows show direction of the movement.

d) Close-up views of the overlay of class B1 (purple, dark blue) and A3 (pink, light blue) structures, showing conformational changes in SNF2H.

e) RMSD of SNF2H between class B2 and B1 (left, B1 shown) structures; B3 and B2 (middle, B2 shown); and B4 and B3 (right, B3 shown) structures. Black arrows show direction of the movement.

f) Close-up views of the overlay of class B4 (color) and B1 (grey) structures, showing conformational changes in SNF2H.

g) RMSD between the class B4 and A1 structures, showing rotation of SNF2H lobes 1 and 2 and brace helix. Class A1 is shown on both views; DNA and histone are in light blue and green, respectively. Black arrows show direction of the movement.

h) ATPase activity of wild-type and mutant SNF2H. ATPase activity of wild type is set to 100.

i) Native polyacrylamide gel stained for DNA showing binding of SNF2H wild-type and mutants to the nucleosome.

j) Native polyacrylamide gel stained for DNA showing nucleosome remodeling by SNF2H wild-type and double mutant R538A R455A.

Group B SNF2H DNA translocation 1

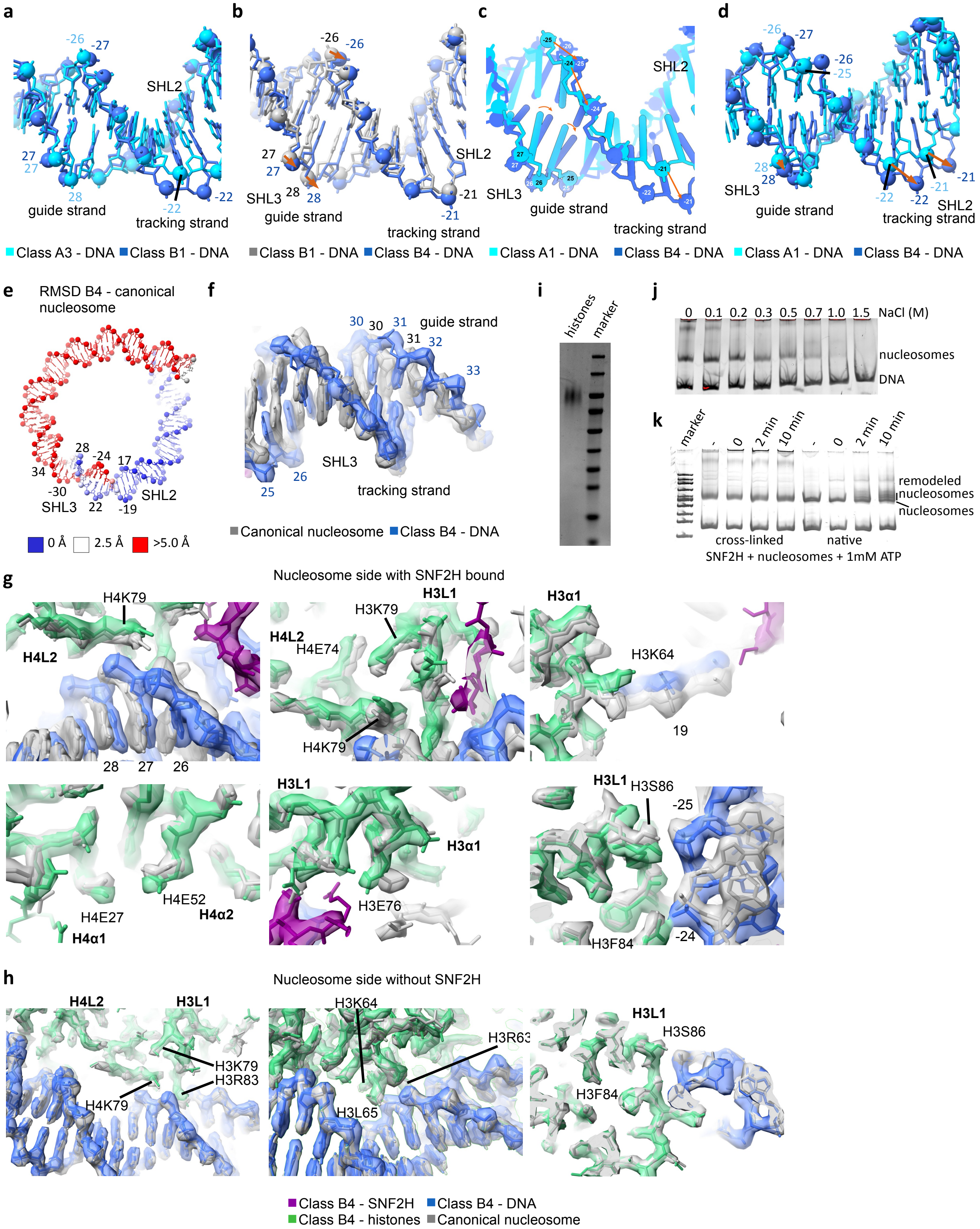

Figure S7. Conformational changes in nucleosome in group B

a) Overlay of the DNA at SHL2 and SHL3 in class B1 (blue) and class A3 (light blue) structures.

b) Overlay of the DNA at SHL2 and SHL3 in class B4 (royal blue) and B1 (grey) structures. Orange arrows show direction of the movement.

c) Cartoon showing overlay of the DNA at SHL2 and SHL3 in class B4 (royal blue) and A1 (light blue) structures. Same view as in Figure 2d. Orange arrows show direction of the movement.

d) Overlay of the DNA at SHL2 and SHL3 in class B4 (royal blue) and A1 (light blue) structures. Orange arrows show direction of the movement.

e) RMSD between the DNA in class B4 and class A1 structures. A1 DNA is shown.

f) Overlay of the DNA at SHL2 and SHL3 in class B4 (royal blue) and canonical nucleosome structures (grey).

g, h) Close-up views of the overlay of class B4 (color) and canonical nucleosome (grey) structures, showing DNA movement and resulting changes to histone residues interacting with DNA at SHL2 and SHL3 on SNF2H-bound side (f) and no changes to DNA or histones on the opposite side of the nucleosome (g).

i) SDS-PAGE showing cross-linked histones migrating at 100 kDa.

j) Native showing with salt-mediated DNA unwrapping from cross-linked nucleosome. These data show that DNA is not cross-linked to histones.

k) Native gel stained for DNA showing remodeling of native (non cross-linked) and cross-linked nucleosomes by SNF2H.

Group C SNF2H DNA translocation 2

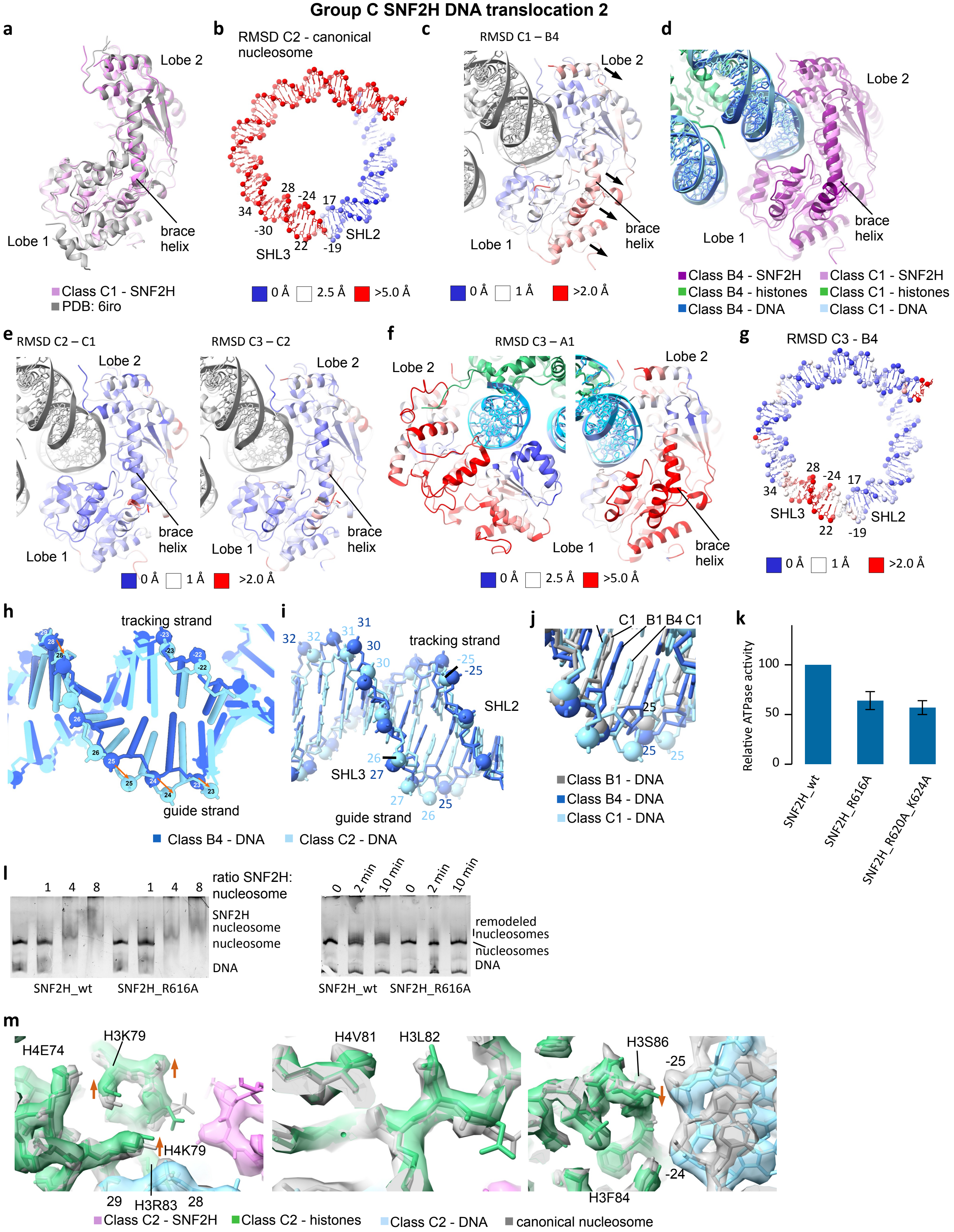

Figure S8. Conformational changes in SNF2H and nucleosome in group C.

a) Overlay of SNF2H in class C1 (violet) and PDB:6iro (grey) structures. SNF2H was used for alignment.

b) RMSD of DNA between class C2 and canonical nucleosome structures, showing DNA movement during translocation. Canonical nucleosome DNA is shown.

c) RMSD of SNF2H between classes C1 and B4 structures. Class B4 is shown. Black arrows show direction of the movement.

d) Close-up views of overlay of class C1 (light colors) and B4 (dark colors) structures, showing conformational changes in SNF2H (pink) and DNA (blue); histones shown in green.

e) RMSD of SNF2H between classes C2 and C1 (left, C1 is shown) and C3 and C2 (right, C2 is shown) structures.

f) RMSD of SNF2H between classes C3 and A1 structures, two views. Class A1 is shown.

g) RMSD of DNA between class C3 and B4 (B4 is shown) structures, showing DNA movement in the second step of DNA translocation.

h) Cartoon showing overlay of the DNA at SHL2 and SHL3 in class C3 (light blue) and B4 (royal blue). Same view as in Figure 3b.

i) Overlay of the DNA at SHL2 and SHL3 in C2 (light blue) and B4 (royal blue) structures.

j) Overlay of the DNA at SHL2 and SHL3 in C1 (light blue), B4 (royal blue) and B1 (grey) structures.

k) ATPase activity of SNF2H wild type and mutants. ATPase activity of wild type is set to 100.

l) Native polyacrylamide gel stained for DNA showing nucleosome remodeling (left) and binding (right) by SNF2H wild type and R616A mutant.

m) Close up views of overlay of class C2 (color) and canonical nucleosome (grey) structures, showing DNA movements and resulting changes in histone residues interacting with DNA at SHL2 and SHL3. Orange arrows show direction of the movement.

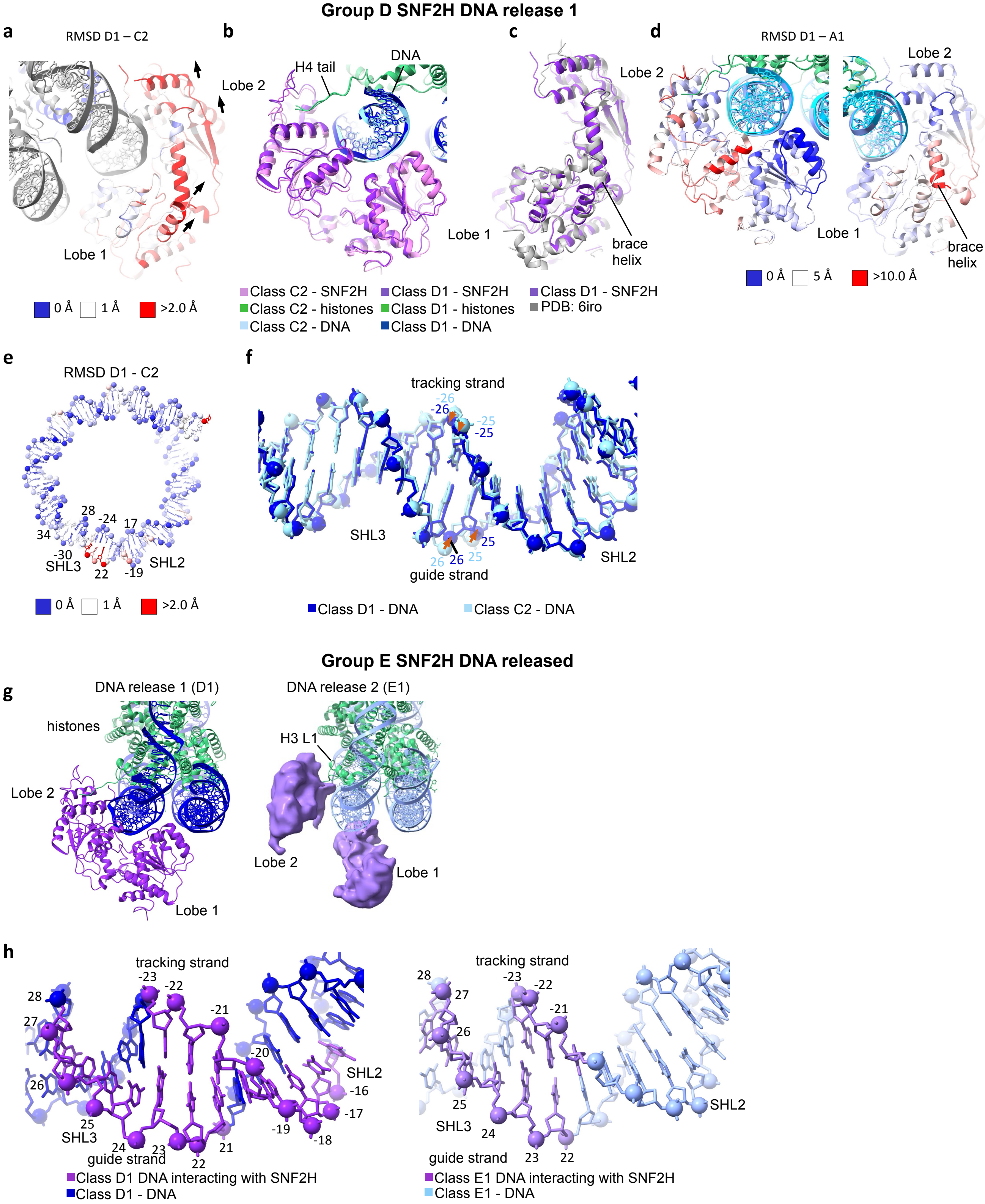

Figure S9. Conformational changes in SNF2H and nucleosome in groups D and E.

a) RMSD of SNF2H between class D1 and C2 structures (C2 is shown). Black arrows show direction

of the movement.

b) Close-up view of overlay of class D1 (dark colors) and C2 (light colors) structures, showing conformational changes in SNF2H. Nucleosome was used for alignment.

c) Close-up view of overlay of SNF2H in class D1 (dark violet) and PDB:6iro structures. SNF2H was used for alignment.

d) RMSD of SNF2H between class D1 and A1 structures (A1 is shown).

e) RMSD of DNA between class D1 and C2 structures, showing DNA movement in the first step of DNA release. DNA from C2 is shown. Orange arrows show direction of the movement.

f) Overlay of the DNA at SHL2 and SHL3 at DNA release step 1 (D1, blue) and DNA translocation step 2 (C2, light blue).

g) Close-up view of class D1 (left) and E1 (right) structures, showing separation of SNF2H lobes 1 and 2 in the latter.

h) DNA at SHL2 and SHL3 in class D1 (left) and E1 (right) structures. For each structure, nucleotides that interact with SNF2H are colored purple; nucleotides that do not interact with SNF2H are colored blue.

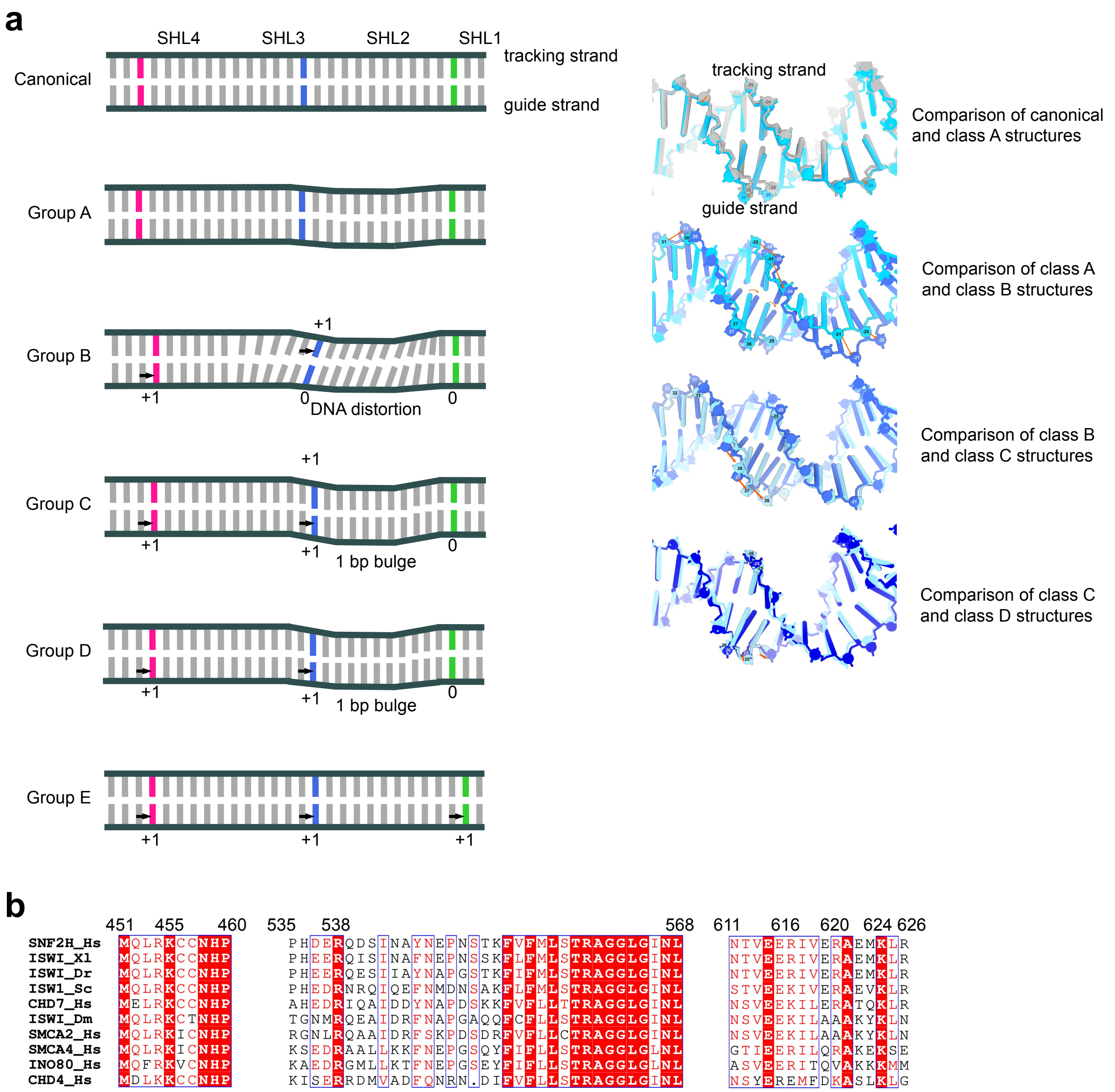

Figure S10. Model for DNA translocation on the nucleosome

a) Schematic model showing DNA translocation on the nucleosome by SNF2H. In Group A DNA is slightly pulled out at SHL2. In Group B, tracking strand is pulled out and both strands move for 1bp until SHL3 (pink bp). At SHL2 and SHL3 only tracking strand moved for 1 nt. We observe that DNA is distorted at SHL2 and SHL3. In group C, both DNA strands are pulled out at SHL2 and both strands move for 1bp until SHL2 (pink and blue bp). DNA adopts canonical conformation with 1bp bulge at SHL2. In group D SNF2H partially releases guide strand.

b) Sequence alignment of motor enzymes from different chromatin remodeling families, showing conservation of key residues that interact with DNA.

Supplementary Information, Table S1: Cryo-EM data collection, refinement, and validation statistics

	A1<br>EMD-xxxx<br>PDB ID xxx	A2<br>EMD- xxxx<br>PDB ID xxx	A3<br>EMD-xxx<br>PDB ID xxx	B1<br>EMD-xxxx<br>PDB ID xxx	B2<br>EMD-xxxx<br>PDB ID xxx	B3<br>EMD- xxxx<br>PDB ID xxx	B4<br>EMD-xxx<br>PDB ID xxx	C1<br>EMD-xxxx<br>PDB ID xxx	C2<br>EMD-xxxx<br>PDB ID xxx	C3<br>EMD- xxxx<br>PDB ID xxx	D1<br>EMD-xxx<br>PDB ID xxx	E1<br>EMD-xxxx<br>PDB ID xxx	E2<br>EMD-xxxx<br>PDB ID xxx	canonical<br>EMD- xxxx<br>PDB ID xxx
**Data collection and rocessing**														
Magnification														
Voltage (kV)	300	300	300	300	300	300	300	300	300	300	300	300	300	300
Electron exposure (e–/Å²)	61.6	61.6	61.6	61.6	61.6	61.6	61.6	61.6	61.6	61.6	61.6	61.6	61.6	61.6
Defocus range (μm)	-1.4 – -3.0	-1.4 – -3.0	-1.4 – -3.0	-1.4 – -3.0	-1.4 – -3.0	-1.4 – -3.0	-1.4 – -3.0	-1.4 – -3.0	-1.4 – -3.0	-1.4 – -3.0	-1.4 – -3.0	-1.4 – -3.0	-1.4 – -3.0	-1.4 – -3.0
Pixel size (Å)	0.6485	0.6485	0.6485	0.6485	0.6485	0.6485	0.6485	0.6485	0.6485	0.6485	0.6485	0.6485	0.6485	0.6485
Symmetry imposed	C1	C1	C1	C1	C1	C1	C1	C1	C1	C1	C1	C1	C1	C1
Initial particle images (no.)	~ 1,000,000	~ 1,000,000	~ 1,000,000	~ 1,000,000	~ 1,000,000	~ 1,000,000	~ 1,000,000	~ 1,000,000	~ 1,000,000	~ 1,000,000	~ 1,000,000	~ 1,000,000	~ 1,000,000	~ 1,000,000
Final particle images (no.)	~ 23, 600	~ 11 600	~ 8100	~ 8300	~ 16 200	~ 33 000	~ 25,500	~ 22,300	~ 30 000	~ 19 000	~ 9,200	~ 7,000	~ 46 000	~ 562000
Map resolution (Å) FSC threshold	3.15	3.25	3.4	3.3	3.25	3.1	3.1	3.2	3.1	3.2	3.4	4.2	3.2	2.6
Map resolution range (Å)														
**Refinement**														
Initial model used	*6WZ5,6NE3*	This study	This study	This study	This study	*6WZ5,6NE3*	This study	This study	This study	*6WZ5,6NE3*	This study	This study	This study	*6WZ5*
Model resolution (Å)	3.15	3.25	3.4	3.3	3.15	3.1	3.1	3.2	3.15	3.2	2.2		3.2	2.6
FSC threshold														
Model resolution range (Å)														
Model composition														
Nonhydrogen atoms	16001	15926	15926	15900	16009	16001	16001	15961	15961	15961	15887			12380
Protein residues	1212	1212	1212	1212	1212	1212	1212	1210	1210	1210	1211			769
Nucleotide	304	300	300	298	304	304	304	303	303	303	299			306
B factors (Å²)														
Protein	189.83	282.2	299.06	277.25	231.49	189.83	201.45	230.45	227.38	225.65	364.23			137.69
Nucleotide	275.69	320.95	334.34	191.69	361.54	275.69	332.65	285.66	270.1	306.03	252.44			220.54
Ligand	182.39	183.97	211.01	195.21	204.43	182.39	153.14	202.17	164.19	165.9	242.37			
R.m.s. deviations														
Bond lengths (Å)	0.004	0.006	0.004	0.005	0.005	0.004	0.006	0.005	0.004	0.005	0.004			0.004
Bond angles (°)	0.617	0.645	0.591	0.771	0.638	0.617	0.725	0.588	0.579	0.6	0.641			0.592
Validation														
MolProbity score	1.82	2.24	2.16	2.16	1.73	1.82	1.95	1.75	1.88	1.96	2.14			1.32
Clashscore	7.75	9.62	10.16	9.83	7.88	7.75	7.61	7.43	8.62	8.41	10.59			5.93
Poor rotamers (%)	1.34	3.44	2.49	2.58	0.96	1.34	1.53	1.15	1.73	1.73	2.3			0
Ramachandran plot														
Favored (%)	95.71	95.29	95.21	95.21	95.63	95.71	94.29	95.71	96.46	95.37	95.37			98.14
Allowed (%)	4.12	4.37	4.45	4.71	4.29	4.12	5.63	4.12	3.45	4.55	4.38			1.86

Supplementary Information, Table S2: List of oligonucleotides used in the study

Oligonucleotides	Sequence
**Primers for SNF2H mutagenesis**	
SNF2H(R616A)_FP	GCGATAGTAGAACGTGCTGAG
SNF2H(R616A)_RP	TTCTTCTACAGTGTTATCAGTTATAAAGC
SNF2H(R620A)_FP	GCGGCTGAGATGAAACTCAGA
SNF2H(R620A)_RP	TTCTACTATTCTTTCTTCTACAGTGTTATC
SNF2H(K624A)_FP	GCGCTCAGACTGGATTCAATAGTC
SNF2H(K624A)_RP	CATCTCAGCACGTTCTACTATTCTTTC
SNF2H(R620A,K624A)_FP	GCGGCTGAGATGGCGCTCAGACTGGATTCAATAGTC
SNF2H(R620A,K624A)_RP	TTCTACTATTCTTTCTTCTACAGTGTTATCAGTTATAAAGCGGAACAC
SNF2H(K455A)_RP	TCTCAACTGCATTAGGATGTT
SNF2H(K455A)_FP	GCGTGTTGTAATCATCCATAT
SNF2H(R538A)_FP	GCGCAAGACTCCATCAATGCA
SNF2H(R538A)_RP	CTCATCATGGGGTGTCTGACCA

Supplementary Information, Table S3: Plasmids used in this study

SJ559	SNF2H(R616A)
SJ560	SNF2H(R620A)
SJ561	SNF2H(K624A)
SJ562	SNF2H(R620A,K624A)
SJ564	SNF2H(K455A)
SJ565	SNF2H(R538A)
SJ566	SNF2H(K455A,R538A)
SJ186	SNF2H(WT)
SJ298	601 widom sequence in pET3a vector

Supplementary Information, Video S1

Video showing motion between classes A-D

Supplementary Information, Video S2

Video showing motion between classes A-D

Supplementary Information, Video S3

Video showing motion starting with ADP-BeF (PDB:8v4y) structure and continuing to classes A-D. The video ends with ADP-BeF bound state (PDB:8v4y).

Supplementary Information, Video S4

Video showing motion starting with ADP-BeF (PDB:8v4y) structure and continuing to classes A-D. The video ends with ADP-BeF bound state (PDB:8v4y).
